# Delayed treatment with an autophagy inhibitor 3-MA alleviates the progression of hyperuricemic nephropathy

**DOI:** 10.1038/s41419-020-2673-z

**Published:** 2020-06-17

**Authors:** Yingfeng Shi, Min Tao, Xiaoyan Ma, Yan Hu, Guansen Huang, Andong Qiu, Shougang Zhuang, Na Liu

**Affiliations:** 1 Department of Nephrology, Shanghai East Hospital, Tongji University School of Medicine, Shanghai, China; 2 School of Life Science and Technology, Advanced Institute of Translational Medicine, Tongji University, Shanghai, China; 3 Department of Medicine, Rhode Island Hospital and Alpert Medical School, Brown University, Providence, RI USA

**Keywords:** End-stage renal disease, Nephrosclerosis

## Abstract

Autophagy is a cell self-renewal process that relies on the degradation of the cytoplasmic proteins or organelles of lysosomes and is associated with development of numerous diseases. However, the therapeutic effect of autophagy inhibition on hyperuricemic nephropathy (HN) and the underlying mechanisms are still unknown. Here, we investigated the effect of delayed treatment with 3-methyladenine (3-MA), a specific autophagy inhibitor, on the development of HN in a rat model. Administration of 3-MA at 21 days following after uric acid injury protected kidney from hyperuricemic-related injuries, as demonstrated by improving renal dysfunction and architecture damage, blocking Beclin-1 and LC3II/I and decreasing the number of autophagic vacuoles. Late treatment with 3-MA was also effective in attenuating renal fibrosis as evidenced by reducing ECM protein deposition, blocking epithelial-to-mesenchymal transition (EMT) and decreasing the number of renal epithelial cells arrested at the G2/M phase of cell cycle. Injury to the kidney resulted in increased expression of TGFβ receptor I, and phosphorylation of Smad3, 3-MA significantly abrogated all these responses. Moreover, inhibition of autophagy suppressed mitochondrial fission, downregulated the expression of Dynamin-related protein 1 (Drp-1), Cofilin and F-actin, and alleviated cell apoptosis. Finally, 3-MA effectively blocked STAT3 and NF-κB phosphorylation and suppressed infiltration of macrophages and lymphocytes as well as release of multiple profibrogenic cytokines/chemokines in the injured kidney. Taken together, these findings indicate that hyperuricemia-induced autophagy is critically involved in the activation of renal fibroblasts, EMT, mitochondrial fission and apoptosis of tubular epithelial cells and development of renal fibrosis. Thus, this study provides evidence for autophagy inhibitors as the treatment of HN patients.

## Introduction

Uric acid is the final product of purine metabolism in human body, and 70% of uric acid is excreted through kidneys^1^. Once uric acid concentration is beyond the physiologycal range, it will cause a variety of pathological responses, such as oxidative stress, mitochondrial dysfunction, apoptosis, and inflammation^1,2^. Epidemiological studies show that increased serum uric acid often precedes the deterioration of chronic kidney disease (CKD)^3^. Moreover, basic studies demonstrate that chronic uric acid injury to the kidney is sufficient to trigger renal tubular damage, interstitial fibrosis, glomerulosclerosis and urate crystal deposition, leading to hyperuricemic nephrology (HN)^1,4,5^. Therefore, further exploration of the mechanism leading to hyperuricemic nephrology will aid in the development of new strategy for treatment of HN.

Tubulointerstitial fibrosis is thought to be the primary pathogenesis for progressive HN^4–6^. Diseased proximal tubules act as a driving force for interstitial fibrosis through activation of autocrine and paracrine signals. A long-term of uric acid exposure causes tubular epithelial cell injury and a maladaptive renal repair with the prolonged cell cycle G2/M arrest^7,8^. Phosphorylation at serine residue (Ser-10) in the histone H3 tail (p-Histone H3) is considered as a hallmark of cells in G2/M phase of cell cycle^9–12^. These tubular cells arrested in G2/M phase facilitate the generation and secretion of multiple profibrotic cytokines, such as TGF-β1 and CTGF^4,7,13^. As autocrine profibrotic signals, they can act on tubular epithelium themselves and induce expression of several transcription factors, including Snail, Twist, and Slug, which drives the transition of renal epithelial cells to mesenchymal phenotype^14^. As paracrine activities on neighboring cells, these tubule-derived molecules can stimulate the resident fibroblasts to produce an excessive amount of extracellular matrix (ECM) proteins. The disequilibrium of ECM modificators such as matrix metalloproteinases (MMPs) will affect depostion of ECM^15,16^. In addition, both hyperuricemia and tubule-secreted cytokines can activate STAT3 and NF-κB-signaling pathways, and induce the infiltration of macrophage and lymphocyte to the injured kidney, leading to aggravation of tubulointerstitial fibrosis^4,5^.

Another important mechanism for hyperuricemia-induced CKD is mitochondrial dysfunction^17,18^. It has been demonstrated that kidney is a high oxygen consumption organ, owing to the intense reabsorption and excretion process that occur in the tubular epithelium, which are very rich in mitochondria. Oxidant urate can induce tubular epithelial dysfunction by increasing mitochondrial superoxide generation^2^. The excessive reactive oxygen species (ROS) generation is injurious to mitochondrial DNA and electron transport chain^19^. In response to the microenvironment, dynamin-related protein 1 (Drp-1) is overexpressed and induces mitochondrial fission to eliminate damaged mitochondria^18,19^. And in the outer membranes of impaired mitochondria, some BCL2 protein family members (i.e., Bax) promote the release of cytochrome *c* to the cytosol to trigger a cascade of caspase-dependent apoptotic signaling to execute apoptosis, leading tubular atrophy and nephron loss^17,18,20^.

Autophagy is an adaptive response. In response to various pathological environments, autophagy is activated to maintain cellular energy homeostasis and clear damaged organelles and misfolded proteins via autolysosomal degradation pathway^21,22^. It is well known that induction of autophagy in proximal tubular cells can be beneficial or detrimental depending on pathological settings. In acute ischemic kidney injury models, autophagy is induced in proximal tubules and plays a protective role^23–25^. However, under sustained stress conditions, such as unilateral ureteral obstruction (UUO), prolonged autophagy in proximal tubules will occur to destroy large proportions of organelles and cytoplasm, leading to an irreversible collapse of cell viability and loss of cytoprotection^26,27^. In agreement with these observations, our recent studies demonstrated that following chronic uric acid injury, autophagy was activated in the tubular epithelial cells and promoted progression of interstitial fibrosis. Inhibition of autophagy by 3-methyladenine (3-MA) was able to protect tubular cells from epithelial–mesenchymal transformation (EMT) and prevent fibrogenesis^5^. 3-MA inhibits autophagy by blocking autophagosome formation and preventing the phase of nucleation^28,29^. However, the underlying mechanism of autophagy inhibition-elicited renoprotection and anti-fibrotic is still not fully elucidated, and the therapeutic effect of 3-MA remains unknown.

The purpose of this study was to assess the therapeutic effect of autophagy inhibition by delayed administration of 3-MA at 21 days, when a certain degree of HN has already occurred and to investigate the mechanisms involved in this process.

## Results

### Delayed administration of 3-MA inhibits autophagy and decreases the number of autophagosome in a rat model of hyperuricemic nephropathy (HN)

**Fig. 1:**
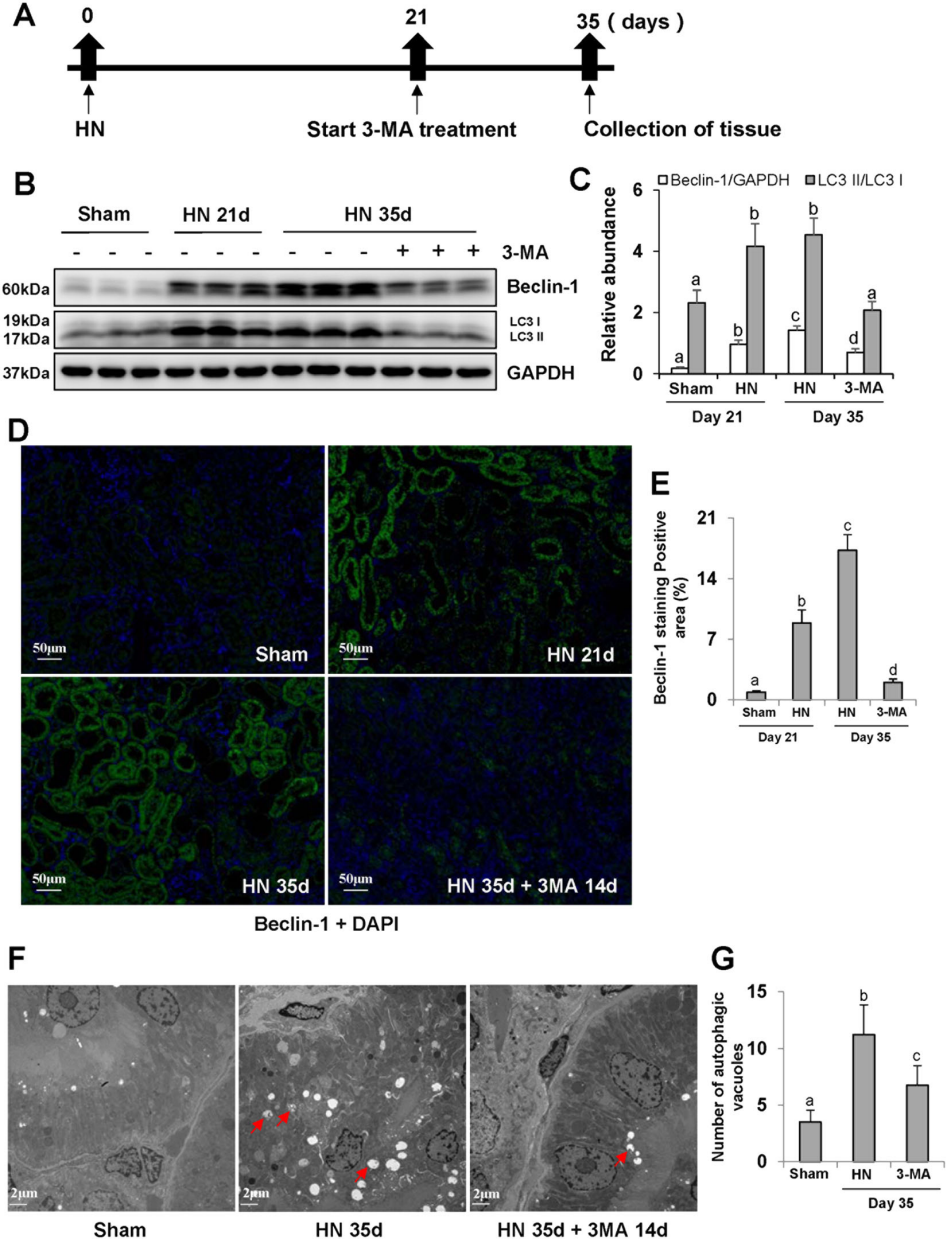
Delayed administration of 3-MA inhibits autophagy and decreases the number of autophagosome in hyperuricemic nephropathy. Schematic experimental design for delayed treatment with 3-MA **a**. The kidney tissue lysates were subjected to immunoblot analysis with specific antibodies against Beclin-1, LC3, and GAPDH **b**. Expression levels of Beclin-1 and LC3II were quantified by densitometry and normalized with GAPDH and LC3I, respectively **c**. Photomicrographs illustrating immunofluorescence co-staining of Beclin-1 and DAPI **d**. The positive area of Beclin-1 was quantitatively analyzed **e**. High magnification of electron micrographs showing autophagosome (red arrows) **f**. Quantitation of the number of autophagosome **g**. Data are represented as the mean ± SEM (*n* = 6). Means with different superscript letters are significantly different from one another (*P* < 0.05). Scale bars in **d** = 50 μm and **f** = 2 μm.

Autophagy has been shown to be involved in a variety of CKDs in animal models, such as UUO^30^, cadmium-induced cytotoxicity^31^, and 5/6 nephrectomy surgery^32^. Here, we examined the effect of late treatment with 3-MA on autophagy in a rat model of HN established by oral administration of a mixture of adenine (0.1 g/kg) and potassium oxonate (1.5 g/kg). 3-MA was given starting 21 days after feeding of adenine and potassium oxonate and then daily for 14 days (Fig. 1a). On days 21 and 35, urine and kidney samples were collected for various analyses.

As shown in Fig. 1b, c, renal autophagy was maintained at a low homeostasis level in normal rats, but it was activated in the hyperuricemic rats, as evidenced by high expression of Beclin-1 and LC3. Delayed treatment with 3-MA remarkably reduced the expression levels of Beclin-1 and LC3. Immunofluorescence staining showed that Beclin-1 was mainly located in renal tubules and highly expressed in the kidney at 21 or 35 days after feeding of adenine and potassium oxonate. 3-MA treatment inhibited its expression (Fig. 1d, e). Transmission electron microscope (TEM) displayed double or multiple membrane structures containing cytoplasm or undigested organelles (Fig. 1f). There were more autophagosomes in the kidney of hyperuricemic rats than control rats. Late treatment with 3-MA substantially reduced the number of autophagosomes in the damaged kidneys (Fig. 1f, g). Taken together, these results indicate that autophagy is activated in the renal tubules of rats with HN and that 3-MA is an effective inhibitor in abrogating autophagy activation under this pathological condition.

### Delayed administration of 3-MA protects against renal dysfunction and tubular injury in hyperuricemic rats

**Fig. 2:**
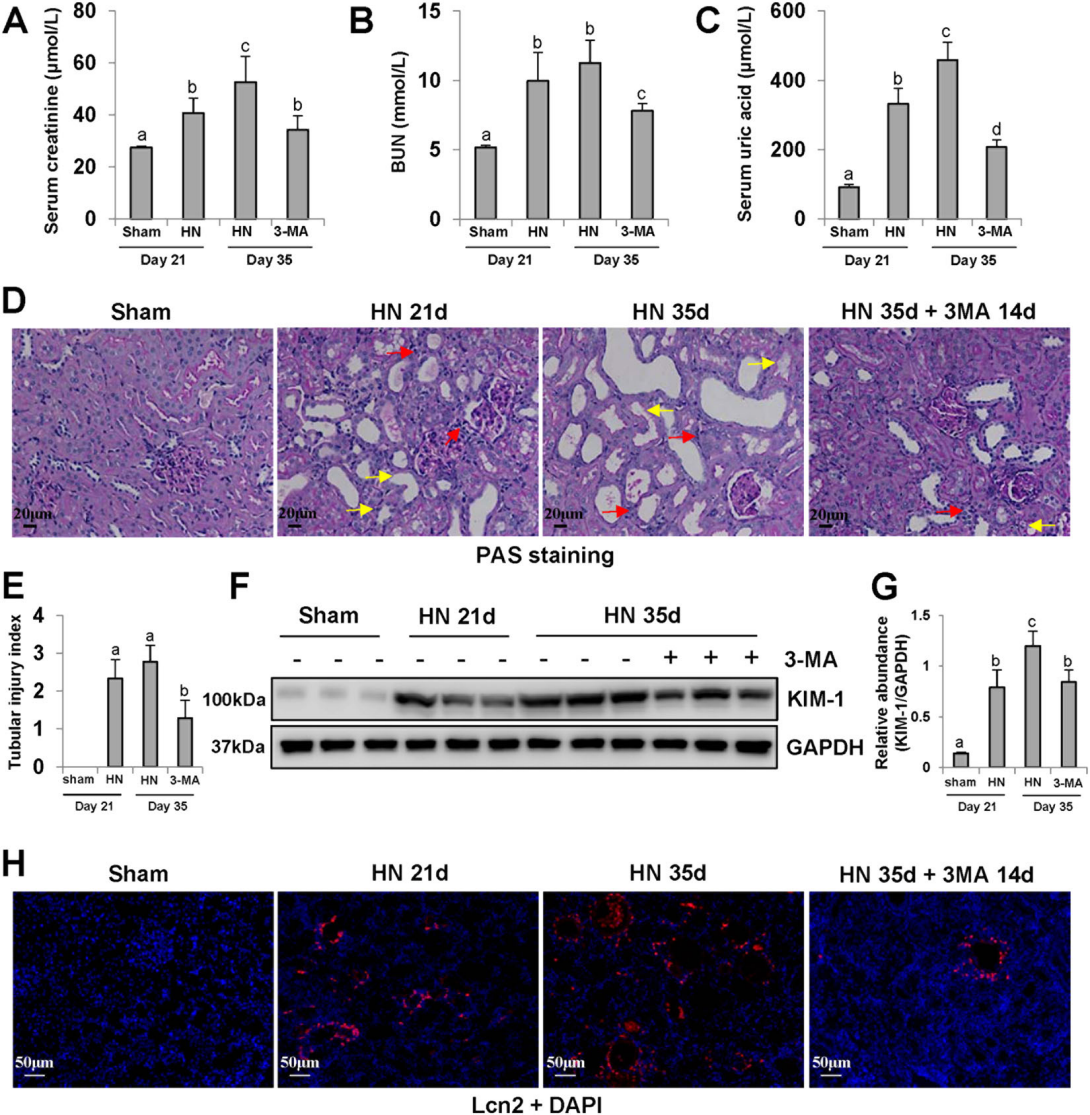
Delayed administration of 3-MA protects against renal dysfunction and tubular injury in hyperuricemic rats. Expression levels of serum creatinine **a**, blood urea nitrogen **b**, and uric acid **c** were examined by using automatic biochemistry assay. Photomicrographs illustrating PAS staining of the kidney tissue. The interstitium was significantly infiltrated with inflammatory cells (red arrows). Proximal tubules were variably atrophic and lined by flat, thin epithelium devoid of brush borders (yellow arrows) **d**. Tubular injury index was conducted according to PAS staining **e**. The kidney tissue lysates were subjected to immunoblot analysis with specific antibodies against KIM-1 and GAPDH **f**. Expression level of KIM-1 was quantified by densitometry and normalized with GAPDH **g**. Photomicrographs illustrating immunofluorescence co-staining of Lcn2 and DAPI **h**. Data are represented as the mean ± SEM (*n* = 6). Means with different superscript letters are significantly different from one another (*P* < 0.05). Scale bars in **d** = 20 μm and **h** = 50 μm.

HN is a common secondary kidney disease, associated with renal dysfunction, tubules destruction, and poor prognosis^33^. In this study, we found that hyperuricemic rats developed renal dysfunction, as indicated in elevated serum creatinine, BUN and serum uric acid, and serious glomerulosclerosis and tubular lesions (Fig. 2a–d). Delayed treatment with 3-MA decreased all these parameters in hyperuricemic rats. Meanwhile, 3-MA showed a protective effect against tubular damage (Fig. 2e) as indicated by reduction of Lcn2 and KIM-1 expressions in the hyperuricemia group. Lcn2 and KIM-1 are two biomarkers in the early stage of renal injury. As expected, a low basal level of KIM-1 was observed in the sham group, and its expression level was increased in HN group at 21 days and further elevated at 35 days after feeding of adenine and potassium oxonate. Blockade of autophagy with 3-MA reduced KIM-1 expression in HN rats after delayed administration for 14 days (from day 21 to 35) (Fig. 2f, g). Immunofluorescence staining of Lcn2 further revealed that Lcn2 was primarily located in renal tubular epithelial cells and highly expressed in injured kidneys (Fig. 2h). Collectively, these data demonstrate that autophagy contributes to renal dysfunction and tubular injury, and delayed treatment of 3-MA is effective in attenuating HN.

### Delayed administration of 3-MA reduces ECM protein deposition and inhibits hyperuricemia-related renal fibrosis

**Fig. 3:**
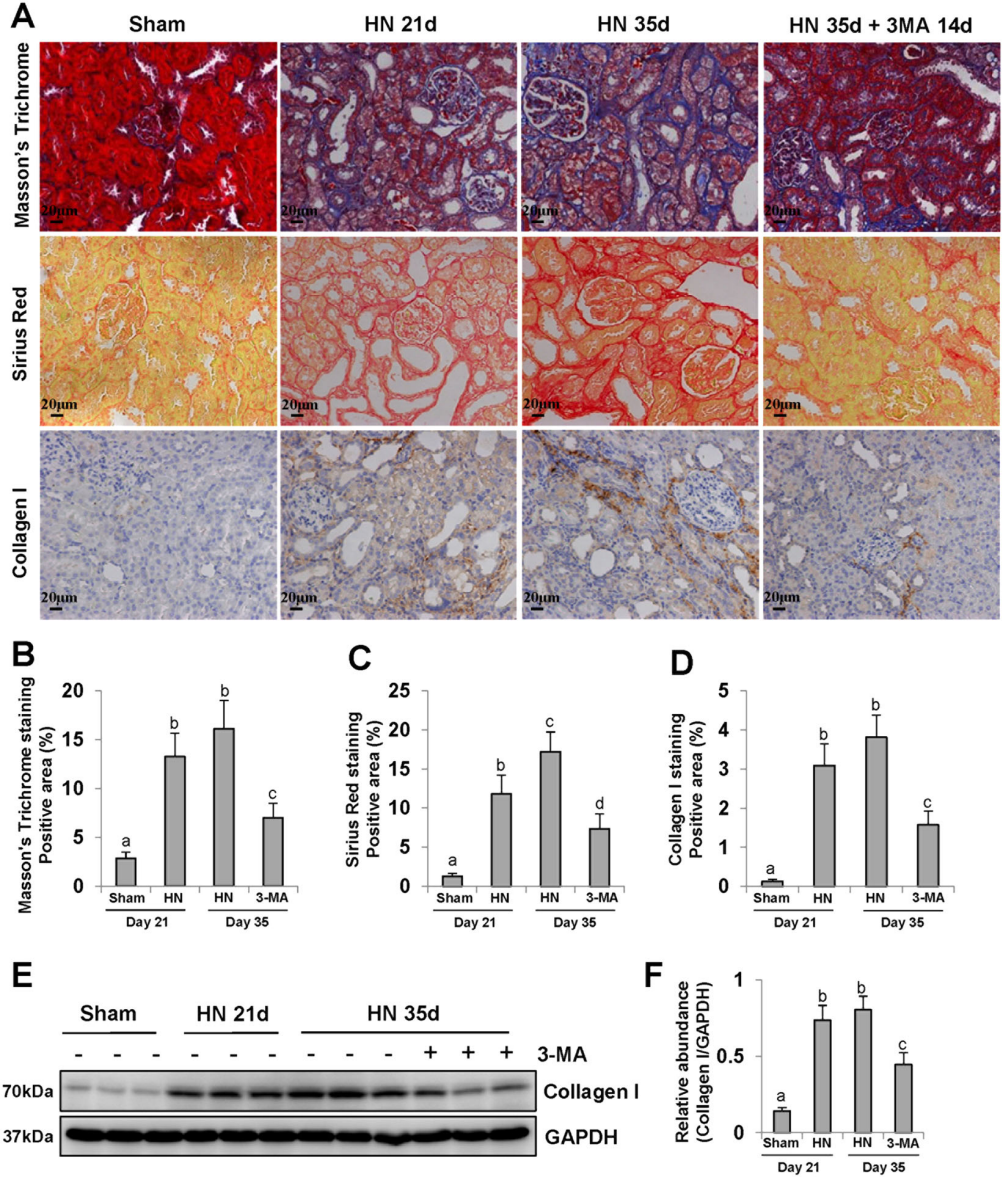
Delayed administration of 3-MA reduces ECM protein deposition and inhibits hyperuricemia-related renal fibrosis. Photomicrographs illustrating Masson’s Trichrome, Sirius Red, and immunohistochemistry with Collagen I **a**. Positive areas of Masson’s Trichrome **b**, Sirius Red **c**, and Collagen I **d** were quantitatively analyzed. The kidney tissue lysates were subjected to immunoblot analysis with specific antibodies against Collagen I and GAPDH **e**. Expression level of Collagen I was quantified by densitometry and normalized with GAPDH **f**. Data are represented as the mean ± SEM (*n* = 6). Means with different superscript letters are significantly different from one another (*P* < 0.05). All scale bars = 20 μm.

Hyperuricemia can cause renal interstitial fibrosis, which eventually leads to end-stage renal disease (ESRD)^34^. As shown in Masson’s trichrome and sirius red staining, there were ECM protein deposition and severe renal interstitial fibrosis in HN kidneys (Fig. 3a). Immunohistochemistry staining also indicated an increase of collagen expression in the kidney of HN rats relative to that in sham rats, especially at 35 days (Fig. 3a). Delayed administration of 3-MA for 14 days effectively inhibited interstitial fibrosis as indicated by reduced staining positive areas (Fig. 3a–d). In line with this observation, immunoblotting results demonstrated that 3-MA was also to impede collagen I overexpression and ECM protein deposition in the kidney of hyperuricemic rats (Fig. 3e, f).

Activity level of MMPs is the key point to maintain the stability of ECM microenvironment^35^. Our results in the HN rat model showed that renal expression levels of MMP2 and MMP9 were up-regulated in both HN groups for 21 and 35 days. Delayed treatment with 3-MA suppressed both MMP2 and MMP9 expressions in varying degrees (Supplementary Fig. 1). Based on these findings, we speculate that inhibition of autophagy with 3-MA may decrease collagen formation and ECM protein deposition through regulating the activity of MMP2/MMP9, which results in alleviating hyperuricemic-related renal fibrosis.

### Inhibition of autophagy with 3-MA blocks renal EMT in the kidney of hyperuricemic rats

**Fig. 4:**
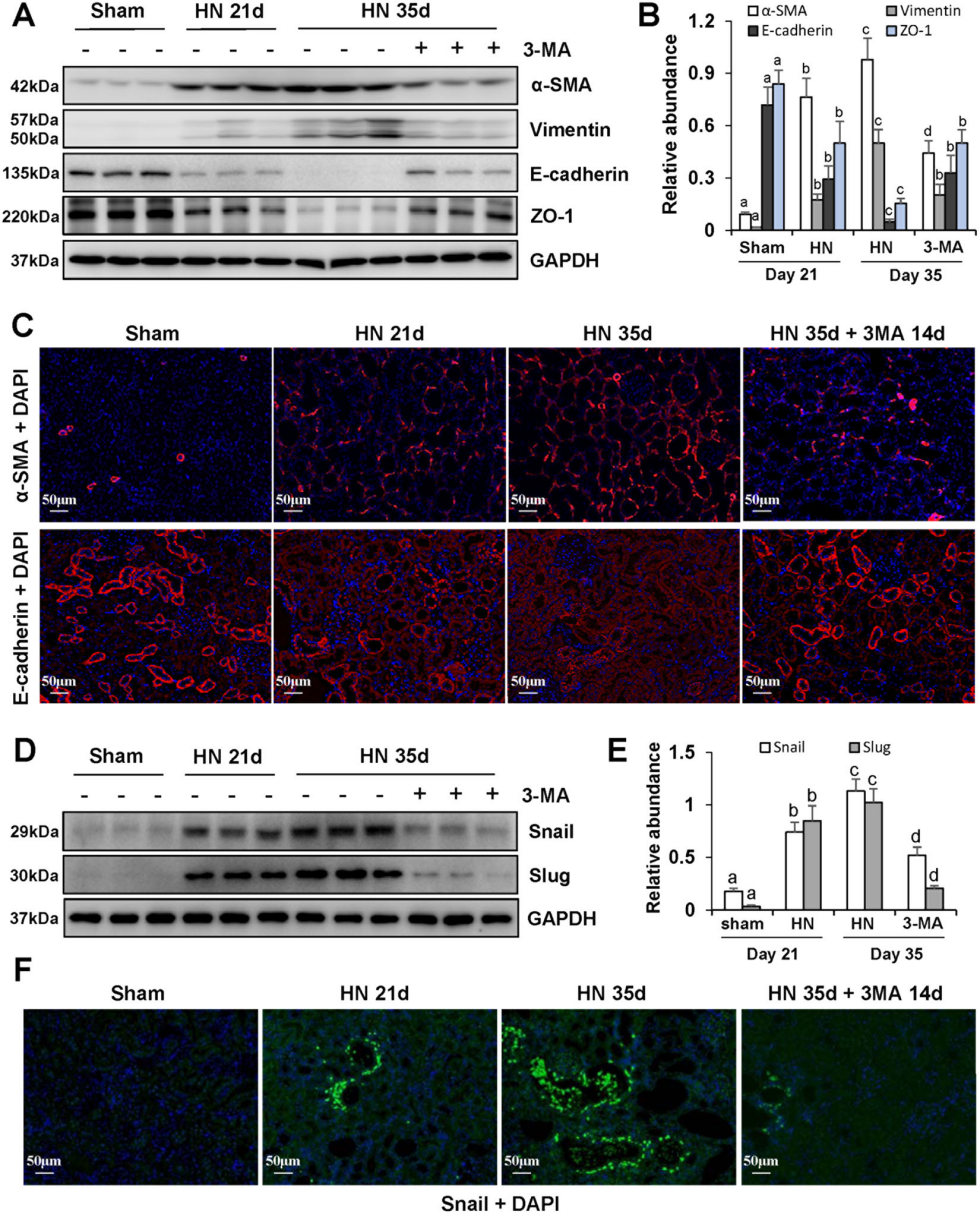
Inhibition of autophagy with 3-MA blocks renal EMT by regulating two transcription factors: Snail and Slug in hyperuricemic rats. The kidney tissue lysates were subjected to immunoblot analysis with specific antibodies against α-SMA, Vimentin, E-cadherin, ZO-1, and GAPDH **a**. Expression levels of α-SMA, Vimentin, E-cadherin, ZO-1 were quantified by densitometry and normalized with GAPDH **b**. Photomicrographs illustrating immunofluorescence of α-SMA and E-cadherin, respectively, costained with DAPI **c**. The kidney tissue lysates were subjected to immunoblot analysis with specific antibodies against Snail, Slug, and GAPDH **d**. Expression levels of Snail and Slug were quantified by densitometry and normalized with GAPDH **e**. Photomicrographs illustrating immunofluorescence co-staining of Snail and DAPI **f**. Data are represented as the mean ± SEM (*n* = 6). Means with different superscript letters are significantly different from one another (*P* < 0.05). All scale bars = 50 μm.

Our previous studies have demonstrated that epithelial-to-mesenchymal transition (EMT) is an important step involved in hyperuricemia-related renal fibrosis^4–6,36^. In this study, we found that 3-MA could prevent EMT development as shown by an increase of epithelial cell markers: E-cadherin and ZO-1, and a decrease of mesenchymal cell markers: vimentin and α-SMA (Fig. 4a, b). At 35 days after hyperuricemic injury, we observed more α-SMA-positive-myofibroblasts located in renal tubulointerstitium, compared with sham rats (Fig. 4c). On the contrary, E-cadherin was mainly observed in tubular epithelial cells and largely expressed in sham kidneys (Fig. 4c). Since Snail and Slug are two pivotal nuclear transcription factors that drive the EMT by suppressing transcription of E-cadherin and other epithelial markers and adhesion molecules^35^, we also examined the effect of 3-MA on their expression. Figure 4d–f shows that treatment with 3-MA dramatically reduced elevated expression of Snail and Slug in the kidney of hyperuricemic mice. Collectively, these data demonstrate that 3-MA inhibits the renal EMT by a mechanism associated with inhibition of nuclear transcription factors in HN.

### 3-MA treatment inhibits G2/M phase cell cycle arrest of renal epithelial cells and TGF-β/Smad3 ignaling pathway

**Fig. 5:**
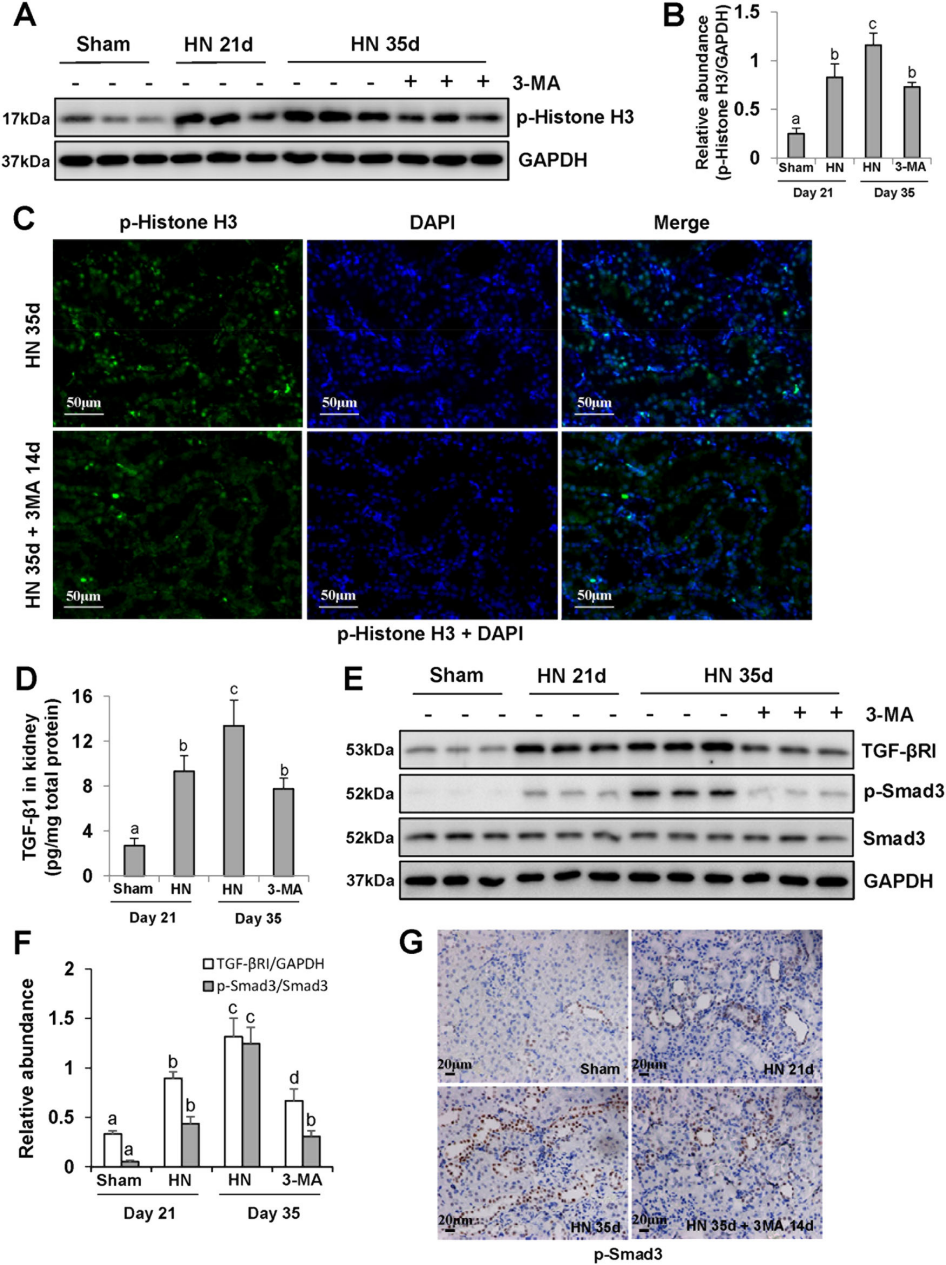
Delayed administration of 3-MA decreases G2/M phase cell cycle arrest of renal epithelial cells and blocks TGF-β/Smad3-signaling pathway. The kidney tissue lysates were subjected to immunoblot analysis with specific antibodies against p-Histone H3 and GAPDH **a**. Expression level of p-Histone H3 was quantified by densitometry and normalized with GAPDH **b**. Photomicrographs illustrating immunofluorescence co-staining of p-Histone H3 and DAPI **c**. Kidney tissue lysates were subjected to the determination of TGF-β1 levels by the ELISA kit **d**. The kidney tissue lysates were subjected to immunoblot analysis with specific antibodies against TGF-βRI, p-Smad3, Smad3, and GAPDH **e**. Expression levels of TGF-βRI and p-Smad3 were quantified by densitometry and normalized with GAPDH and Smad3, respectively **f**. Photomicrographs illustrating immunohistochemistry staining of p-Smad3 **g**. Data are represented as the mean ± SEM (*n* = 6). Means with different superscript letters are significantly different from one another (*P* < 0.05). Scale bars in **c** = 50 μm and **g** = 20 μm.

The renal tubular epithelial cells arrested at G2/M phase of cell cycle are a profibrotic phenotype that acquires the ability to overproduce TGF-β1 and other profibrotic factors^37,38^. Thus, we examined the expression of phospho-Histone H3 at serine residue 10 (p-Histone H3), a hallmark of G2/M phase arrest^9–12^, in each group. As shown in Fig. 5a, b, renal expression level of p-Histone H3 in HN group was higher than that in sham rats, whereas delayed treatment with 3-MA decreased the number of p-Histone H3-positive renal tubular epithelial cells in injured kidneys of HN rats (Fig. 5c). In parallel, 3-MA reduced TGF-β1 expression in the kidney of HN rats (Fig. 5d). In addition, immunoblot analysis also confirmed that delayed administration of 3-MA reduced renal expression of TGF-βRI and phosphorylation of Smad3 in hyperuricemic rats, but had no impact on the expression of total Smad3 (Fig. 5e, f). Immunohistochemistry of p-Smad3 demonstrated that p-Smad3 was predominantly localized in tubular epithelial cells in injured kidneys associated with HN, and 3-MA reduced the number of p-Smad3-positive cells (Fig. 5g). As such, these data reveal that 3-MA treatment has the ability to decrease the number of renal epithelial cells arrested at G2/M phase cell cycle and abrogate the TGF-β/Smad3-signaling pathway in hyperuricemic rats.

### Blockade of autophagy alleviates mitochondrial fission in hyperuricemic rats

**Fig. 6:**
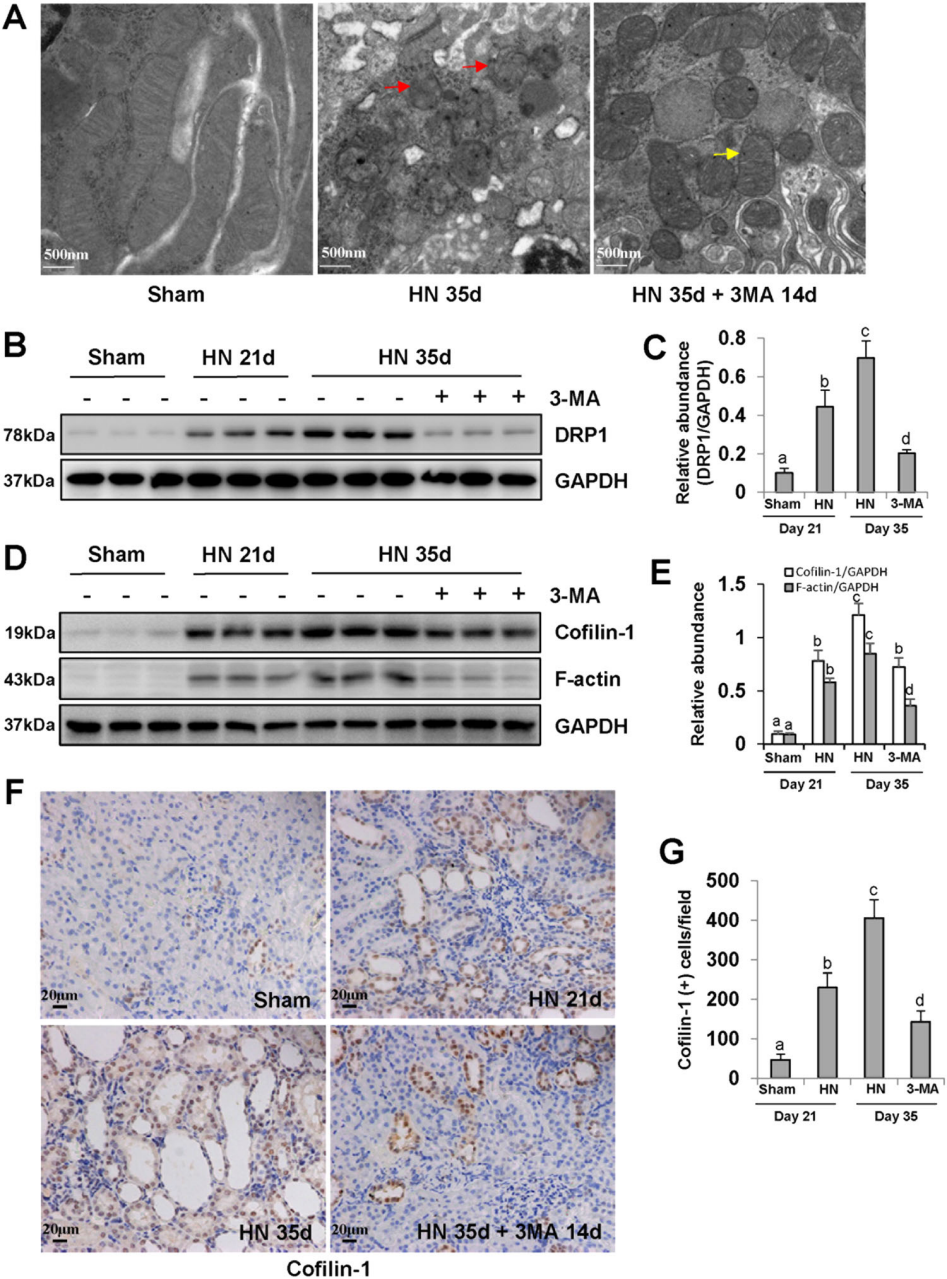
Blockade of autophagy alleviates mitochondrial fission in hyperuricemic rats. High magnification of electron micrographs of kidneys (red arrow represents damaged mitochondria and yellow arrow represents mild pathological mitochondria) **a**. The kidney tissue lysates were subjected to immunoblot analysis with specific antibodies against DRP1 and GAPDH **b**. Expression level of DRP1 was quantified by densitometry and normalized with GAPDH **c**. The kidney tissue lysates were subjected to immunoblot analysis with specific antibodies against Cofilin-1, F-actin, and GAPDH **d**. Expression levels of Cofilin-1 and F-actin were quantified by densitometry and normalized with GAPDH **e**. Photomicrographs illustrating immunohistochemistry staining of Cofilin-1 **f**. Quantitation of the number of Cofilin-1 positive cells **g**. Data are represented as the mean ± SEM (*n* = 6). Means with different superscript letters are significantly different from one another (*P* < 0.05). Scale bars in **a** = 500 nm and **f** = 20 μm.

Several reports have demonstrated that mitochondrial dysfunction contributes to both acute and CKDs^39–42^. By utilizing TEM, we examined the mitochondrial fission in renal tubular epithelial cells. Mitochondrial fission in tubular epithelial cells from HN kidneys was characterized by swelling and shortening of mitochondria, and disappearance of mitochondrial cristae (Fig. 6a). Delayed administration of 3-MA inhibited expression of Drp1, a key protein which combined with F-actin to mediate mitochondrial fission^19,43^ (Fig. 6b, c). 3-MA treatment also had inhibitory effect on Cofilin-1, an actin-depolymerizing factor that can increase actin filament turnover and promote mitochondrial fission^43^ (Fig. 6d, e). Cofilin-1-positive cells were mainly located in the damaged tubular epithelial cells (Fig. 6f, g). These data suggest that blockade of autophagy with 3-MA is able to maintain mitochondrial homeostasis in hyperuricemic rats.

### Delayed administration of 3-MA inhibits renal tubular epithelial cells apoptosis in hyperuricemic rats

**Fig. 7:**
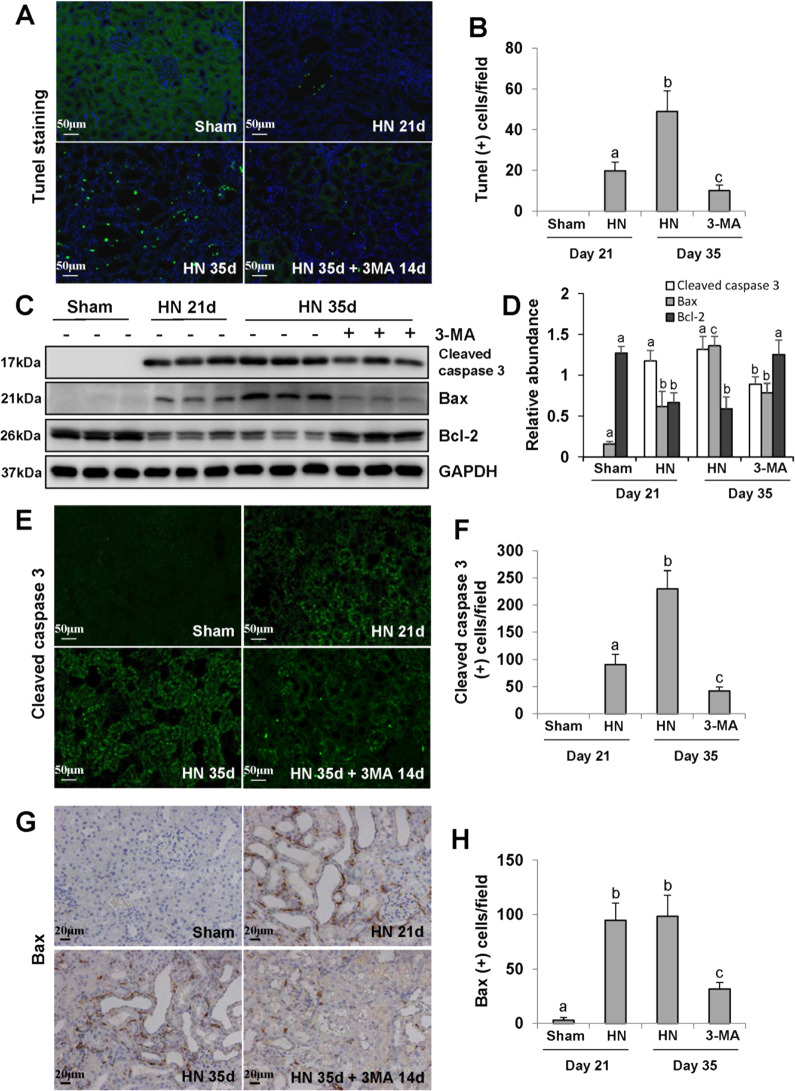
Delayed administration of 3-MA abrogates renal tubular epithelial cells apoptosis in hyperuricemic rats. Photomicrographs illustrating Tunel staining of the kidneys **a**. Quantitation of the number of Tunel-positive cells **b**. The kidney tissue lysates were subjected to immunoblot analysis with specific antibodies against Cleaved caspase 3, Bax, Bcl-2, and GAPDH **c**. Expression levels of Cleaved caspase 3, Bax, and Bcl-2 were quantified by densitometry and normalized with GAPDH **d**. Photomicrographs illustrating immunofluorescence staining of Cleaved caspase 3 **e**. Quantitation of the number of Cleaved caspase 3 positive cells **f**. Photomicrographs illustrating immunohistochemistry staining of Bax **g**. Quantitation of the number of Bax-positive cells **h**. Data are represented as the mean ± SEM (*n* = 6). Means with different superscript letters are significantly different from one another (*P* < 0.05). Scale bars in **a**, **e** = 50 μm and **g** = 20 μm.

Mitochondrial dysfunction is associated with increased mitochondrial permeability, release of cytochrome *c*, activation of caspase-3, and regulation of Bax/Bcl-2 family proteins^44,45^. We hypothesize that 3-MA may inhibit renal tubular epithelial cell apoptosis through inhibiting mitochondrial fission. TUNEL staining showed more apoptotic tubular epithelial cells in the kidney of HN rats compared with the sham control group. Delayed administration of 3-MA reduced the number of TUNEL(+) cells in injured kidneys (Fig. 7a, b). 3-MA treatment also decreased expression levels of cleaved caspase 3 and Bax, and restored expression of Bcl-2 in 35 days-HN group (Fig. 7c, d). Immunohistochemistry and immunofluorescence staining pointed out that cleaved caspase 3 and Bax were mainly expressed in the damaged tubular epithelial cells, and significantly decreased after 3-MA treatment (Fig. 7e–h). Taken together, mitochondrial fission induces renal tubular epithelial cells apoptosis in hyperuricemic kidney, while 3-MA maintains mitochondrial homeostasis so as to prevent cell apoptosis and protect the integrity of structure and function of renal tubules.

### 3-MA suppresses activation of STAT3 and NF-κB-signaling pathway and infiltration of macrophages and lymphocytes in the kidney of hyperuricemic rats

**Fig. 8:**
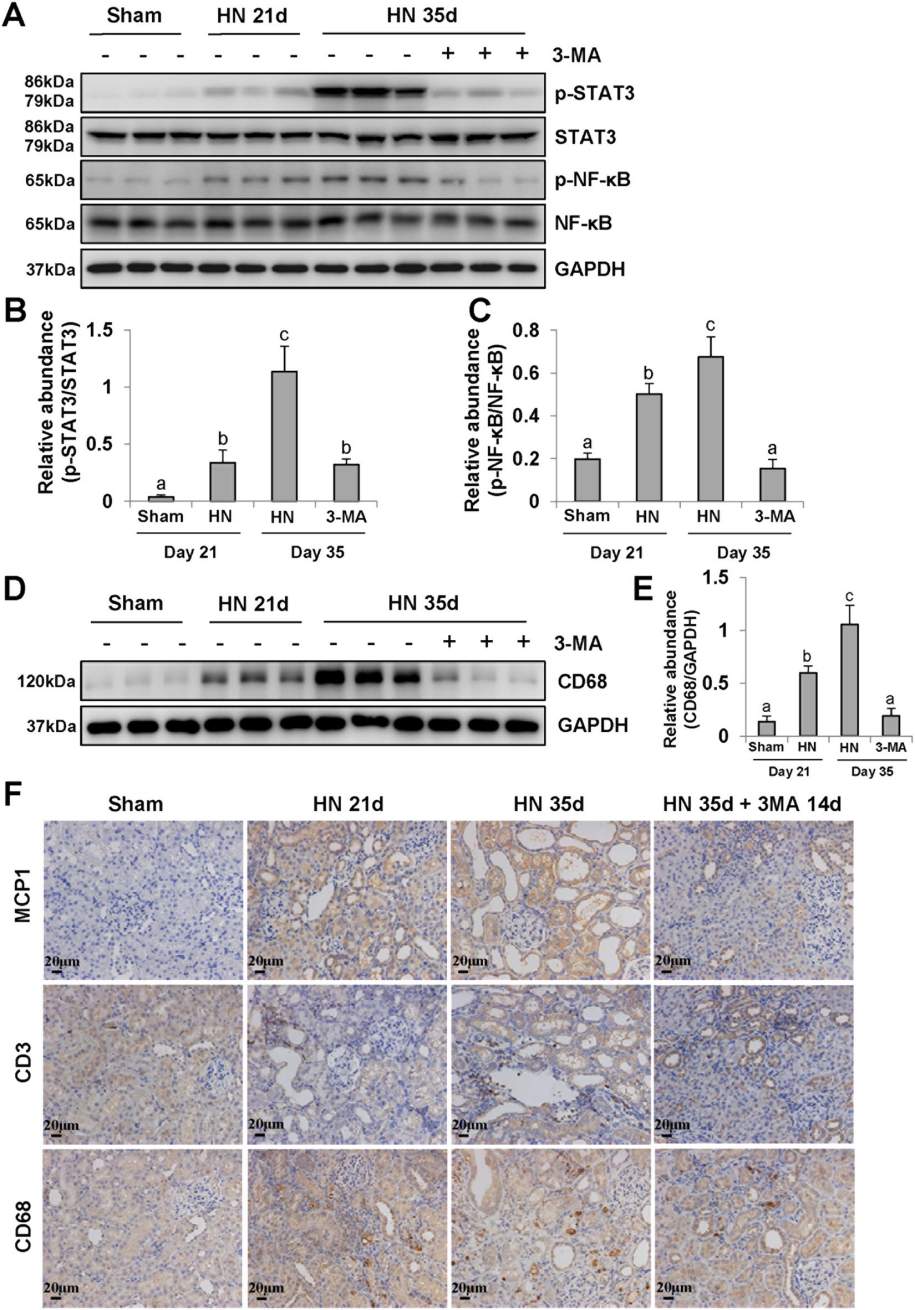
Delayed administration of 3-MA blocks inflammation responses in the kidney of hyperuricemic rats. The kidney tissue lysates were subjected to immunoblot analysis with specific antibodies against p-STAT3, STAT3, p-NF-κB, NF-κB, and GAPDH **a**. Expression level of p-STAT3 was quantified by densitometry and normalized with STAT3 **b**. Expression level of p-NF-κB was quantified by densitometry and normalized with NF-κB **c**. The kidney tissue lysates were subjected to immunoblot analysis with specific antibodies against CD68 and GAPDH **d**. Expression level of CD68 was quantified by densitometry and normalized with GAPDH **e**. Photomicrographs illustrating immunohistochemistry staining of MCP1, CD3, CD68 **f**. Data are represented as the mean ± SEM (*n* = 6). Means with different superscript letters are significantly different from one another (*P* < 0.05). All scale bars = 20 μm.

We and other research teams have revealed that inflammation is critically involved in the pathogenesis of HN^4,5,46,47^. As shown in Fig. 8a, phosphorylation of STAT3 and NF-κB, two key signaling molecules associated with inflammatory response, were increased in the kidney of HN rats compared with that in sham rats. Delayed administration of 3-MA inhibited their phosphorylation but did not alter expression of their total protein levels (Fig. 8a–c). We further validated the inhibitory effect of 3-MA on macrophage infiltration. The basal level of CD68 was negligible in the kidney tissue of normal rats, but it was remarkably upregulated in the HN groups at 21 and 35 days after hyperuricemic injury. Treatment with 3-MA reduced CD68 expression to the basal level (Fig. 8d, e). Moreover, immunohistochemistry staining confirmed the anti-inflammatory effect of 3-MA in injured kidney, which was coincident with the reduction of monocyte chemoattractant protein-1 (MCP-1), CD3-positive lymphocytes and CD68-positive macrophage infiltration (Fig. 8f). Therefore, 3-MA is able to improve the inflammatory response and microenvironment in HN kidneys. Eventually, we summarized the detailed mechanisms by which autophagy regulates hyperuricemia-related renal injury (Supplementary Fig. 2).

## Discussion

Our previous study has provided evidence for preventative renal protection of autophagy inhibition in HN^5^. However, we are curious whether autophagy inhibition also produces a therapeutic effect on already formed renal injury, which will have more value for clinical HN patients. Thus, we further examined the therapeutic effect of autophagy inhibition and the underlying mechanism in a rat model of HN by administration of an autophagy inhibitor 3-MA starting at 21 days after feeding of adenine and potassium oxonate in this present study. Our results showed that late treatment with 3-MA significantly inhibited uric acid-induced renal interstitial fibrosis by inhibiting EMT, reducing the number of tubular cells arrested at the G2/M phase of cell cycle, and reducing ECM proteins accumulation. 3-MA also ameliorated tubular cell atrophy and death by restoring mitochondrial homeostasis and decreasing damaged mitochondrial-elicited apoptosis. Moreover, 3-MA attenuated inflammation responses and activation of STAT3 and NF-κB-signaling pathways. Taken together, autophagy inhibition is the main mechanism for 3-MA-elicited a series of renoprotective effects. Delayed treatment with 3-MA is effective in attenuating the progression of renal fibrosis in HN.

Autophagy is a dynamic process associated with balancing intracellular energy and resource. In addition to turnover of organelles and proteins, autophagy can influence a series of other cellular processes, including metabolic pathway, cell cycle and death, inflammatory and immune responses^21,48,49^. The role of autophagy in the pathogenesis of disease is complicated and involved in both physiological or pathological regulation. Compared with the role of adaptive repair of autophagy after acute stimulus^23,24,50,51^, a maladaptive outcome more likely appears in the tubular epithelium following the repeated and prolonged injury^26,52^. Specifically, our study observed increased autophagosome numbers in tubular epithelium accompanied by cumulative ECM in the interstitium during chronic uric acid stimulation. While delayed treatment by autophagy inhibition rescued tubular epithelial cells from G2/M arrest, overturned EMT, and suppressed tubulointerstitial fibroblasts activation. These observations, along with our previous study, suggest a propathogenic role of autophagy in HN^5^.

In this mechanism, we speculated that autophagy inhibition alleviates tubular cells arrested at G2/M phase of cell cycle and reduces tubular cell-released profibrotic cytokines, such as TGF-β1. Given the autocrine and paracrine roles of tubular cell-drived profibrotic cytokines, their reduction not only weakens the EMT of tubular epithelium itself, but also reduces the activation of neighboring fibroblasts. In fact, 3-MA not only inhibits class I phosphoinostitide 3-kinase, but also suppresses class III phosphoinostitide 3-kinase. Thus, it is reasonable to speculate that 3-MA may have the potential to regulate other signaling pathways in addition to its role in mitophagy. In this regard, we observed that 3-MA can inhibit expression of two key transcription factors Snail or Slug that drive the EMT process. Meanwhile, both previous and current presented studies showed the inhibitory effect of 3-MA on TGF-β1/Smad3-signaling pathway, as the upstream regulator of Snail and Slug. We consider TGF-β1/Smad3-mediated EMT transcription factors as the key point for preventative or therapeutic 3-MA administration to inhibit EMT, thus blocking renal fibrosis. Further elucidation of potential non-autophagic pathways affected by 3-MA is worth to explore in the future.

It has been demonstrated that mitochondria is the major energy-producing organelle of the proximal tubular cells^18^. Hyperuricemia induces tubular epithelial cell dysfunction by increasing mitochondrial superoxide generation and triggering oxidative stress^6,53^. Thus, dynamic mitochondrial network shifts towards mitochondrial fission to remove damaged mitochondria. In mammals, mitochondrial fission involves cytoplasmic protein, Drp-1, which interacts with cellular cytoskeletal actin filaments to form a ring structure to encircle and constrict at a site on the outer mitochondrial membrane^18,43^. While actin-depolymerizing factor Cofilin accelerates both actin assembly and disassembly, increases actin filament turnover and thus exacerbates mitochondrial fragmentation^43^. We observed that mitochondrial fission in tubular epithelial cell occurs as early as within 3 weeks after HN in vivo, evidenced by increased expression of Drp1, Cofilin-1, and F-actin. After urate injury, some slightly damaged mitochondrial fragmentations tend to fusion and repair their function by complementation. Whereas others may refuse into long filamentous mitochondria, which is encased by lysosomes and triggered mitophagy process. Our electron micrographs of kidney tissue showed an integrated oval outer mitochondrial membrane and clear cristae formed by inner mitochondrial membrane in sham group. After sustained 5 weeks of uric acid exposure, the number of mitochondria decreased significantly. Damaged mitochondria fissured and generated smaller and spherical mitochondria. Simultaneously mitolysosomes were formed and identified as single membrane structures containing undigested mitochondrial fragmentation. While delayed treatment by an autophagy inhibitor for 14 days effectively inhibited the mitophagy process, partly reduced mitochondrial fission, and shifted dynamics towards fusion and repair. Although accumulating evidence emphasizes a protective effect of mitophagy through removing oxidative stress in the cases of AKI (such as ischemic preconditioning and cisplatin nephrotoxicity)^23,24,50^, emerging views implicate mitophagy as a possible effector of cell death programs^54,55^. Thus, our study suggests a propathogenic role for sustaining mitophagy and general autophagy in HN.

It is well known that one of the characteristic pathology of CKD is the tubular atrophy with flat and thin epithelium devoid of brush borders, even loss of nephron^4,56^. During the sustained urate injury, the protective effect of mitochondrial fission and autophagy may turn into a vicious circle of excessive loss of organelles or cytoplasm which attributed to overblow apoptosis or necroptosis signal^54^. The mechanisms by which mitochondria induces and mediates the process of cell death often involves the permeabilization of mitochondrial membrane^18^. The BCL2 protein family release cytochrome *c* to the cytosol to trigger a cascade of apoptotic signaling to execute apoptosis^17,18,20^. Notablely, 3-MA is an effective autophagy inhibitor by blocking autophagy vesicle initiation-related phosphoinositide 3-kinase (PI3K)/protein kinase B (AKT)/mammalian target of rapamycin1 (mTOR) pathway^57^. Recent research also reported that mTOR is responsible for mitochondrial fission by stimulating the translation of mitochondrial fission process 1 (MTFP1)^58^. Therefore, it is reasonable to speculate that 3-MA treatment exerts a renoprotective effect by reducing the mitochondrial fission, mitophagy, and their induced apoptosis. Breaking the vicious circle of mitochondria would help to retain the number of mitochondria and ATP production, which may increase the vitality of urate excretion transporter and in turn facilitate urate clearance.

Generally, uric acid in combination with damaged mitochondria-released ROS is thought to be prime criminals for triggering inflammatory response in HN^2,4^. Emerging evidence showed that autophagy protects kidney tubules from acute pathological stimuli through removing ROS-producing mitochondria^23,48^. However, dysregulated autophagy caused by chronic prolonged injury, often coexists with inflammation to promote tubule epithelium atrophy and interstitial fibrosis^5,23^. Moreover, our previous and present study suggested that autophagy inhibition helps to alleviate inflammatory response^5^. On the one hand, 3-MA increases the clearance of inflammatory stimulus urate through partly increasing the number of mitochondria and ATP production and thus improving the vitality of urate transporter. On the other hand, as a PI3K inhibitor, 3-MA indirectly blocks activation of its downstream AKT, partly suppressing inflammatory signaling pathway transduction^59^. This hypothesis is supported by our electron microscope observation that reserved number of mitochondria was accompanied by decreased serum uric acid in 3-MA treatment group, as well as by our immunoblotting results that the reduced expression of STAT3 and NF-κB, and immunohistochemical finding that the attenuation of infiltration of macrophage and lymphocyte after 3-MA treatment.

Compared with our previous study^5^, this present research has a variety of advantages and novel findings in mechanisms of autophagy involved in HN. Firstly, this study proves that autophagy inhibition also presents a therapeutic effect on already formed renal injury, which is more meaningful than simple preventative effect supported by our previous studies, and more practical for clinical HN patients. Secondly, we reveal that blockade of autophagy can remarkably inhibit renal EMT by downregulation of two EMT-related nuclear transcription factors (Snail and Slug). However, it is no touch in this mechanism in our previous study^5^. Thirdly, inhibition of autophagy suppressed mitochondrial fission, downregulated the expression of Drp-1, Cofilin, and F-actin and alleviated cell apoptosis. It is also the novel finding in this present study than our previous publication. Combination with two parts of research suggests inhibition of autophagy plays the beneficial role in both preventative and therapeutic effect in HN injury.

In summary, our data showed a propathogenic role of autophagy for HN and that delayed treatment with an autophagy inhibitor 3-MA exerts an antifibrotic effect to kidney through a variety of mechanisms, including reduction of tubular cells G2/M arrest, EMT, and ECM accumulation, recovery of mitochondrial homeostasis as well as attenuation of apoptosis and inflammation. Therefore, autophagy inhibitor 3-MA may hold a therapeutic potential for treatment of patients with HN.

## Materials and methods

### Antibodies and reagents

3-MA was purchased from Selleckchem (Houston, TX). Antibodies to Beclin-1, Snail, p-Smad3, Smad3, Vimentin, E-cadherin, Drp1, Cleaved caspase 3, p-STAT3, and STAT3 were purchased from Cell Signaling Technology (Danvers, MA). Antibodies to Collagen I (A2), Cofilin-1, p-NF-κB, NF-κB, GAPDH, TGF-βRI, CD68, and CD3 were purchased from Santa Cruz Biotechnology, Inc. (Santa Cruz, CA). Antibodies to p-Histone H3, Slug, ZO-1, Bcl-2, MCP1, MMP2, MMP9, and F-actin were purchased from Abcam (Cambridge, MA). Antibody to LC3 was purchased from Novus Biologicals (Littleton, CO). Antibodies to Lcn2 and KIM-1 were purchased from R&D System (Minneapolis, MN). Antibody to Bax was purchased from BD Bioscience (San Diego, CA). TGF-β1 ELISA kit was purchased from R&D systems (Minneapolis, MN). Antibody to α-smooth muscle actin (α-SMA), secondary antibodies for Western blot, adenine, potassium oxonate, and all other chemicals were purchased from Sigma (St. Louis, MO).

### Animals and treatment

Male Sprague-Dawley rats (Shanghai Super-B&K laboratory animal Corp. Ltd) that weighed 200–220 g were housed under a 12:12-h light–dark cycle with food and water supplied ad libitum at the Experimental Animal Center of Tongji University. All rats were acclimated to this environment for 7 days before experiments. The HN rat model was established as described in our previous study^4,36^. Briefly, a mixture of adenine (0.1 g/kg) and potassium oxonate (1.5 g/kg) dissolved in distilled water was administered via P.O. once daily for 5 weeks. To examine the therapeutic effect on established renal injury, 3-MA (15 mg/kg) in saline was administered intraperitoneally starting after 21 days and given daily for 14 days to HN rats. Autophagy inhibition by 3-MA with dosage of 15 mg/kg/d i.p. is consistent with studies in the rat models^60,61^. The sham group was injected with an equal volume of saline as a control. Twenty-four male rats were randomly assigned to four groups with six rats in each group: sham, HN for 21 days, HN for 35 days, and HN for 21 days delayed treatment with 3-MA for 14 days. After 5 weeks, the animals were sacrificed and the kidney samples were collected for protein analysis and histological examination. Blood was taken for the measurement of serum uric acid, blood urea nitrogen (BUN), and creatinine. The animal protocol was reviewed and approved by the Institutional Animal Care and Use Committee at Tongji University (Shanghai, China).

### Renal function assay

Levels of serum uric acid, serum creatinine, and BUN were examined by automatic biochemistry assay (P800, Modular, USA).

### ELISA analysis

ELISA detection of TGF-β1 was performed in accordance with the manufacturer’s instructions.

### Transmission electron microscope

TEM was performed to observe the morphology of autophagosome and mitochondria. After indicated treatments, the rats were killed and perfused with 10 ml (10 units/ml) heparin, followed by 50 ml fixative. Kidneys were then harvested and fixed in the same fixative (100 mmol/l sodium cacodylate, 2 mmol/l CaCl_2_, 4 mmol/l MgSO_4_, 4% paraformaldehyde, and 2.5% glutaraldehyde). Approximately 1 mm^3^ of tissue cube was collected from each kidney, including a portion of renal cortex and outer medulla for standard TEM processing. And various autophagic structures including phagophore, autophagosome, and autolysosome in renal tubular epithelial cells were revealed at high magnification (×5000). For quantitation, 20–30 fields of magnification (×5000) were randomly selected from each kidney and digital images with scale bars were taken. Using Axio Vision 4 software, the amount of autophagic vacuoles per unit cytoplasmic area of 100 μm was evaluated.

### Immunoblot analysis

Immunoblot analysis of kidney samples from each group was conducted as described previously^62^. The densitometry analysis of immunoblot results was conducted by using NIH Image software (National Institutes of Health, Bethesda, MD).

### Immunohistochemical and immunofluorescent staining

Formalin-fixed kidneys were embedded in paraffin and prepared in 3-μm-thick sections. For assessment of renal fibrosis, Masson’s Trichrome staining and Sirius red staining were carried out according to the protocol provided by the manufacturer (Sigma, St. Louis, MO). Immunohistochemical and immunofluorescent staining were performed according to procedures described in our previous studies^4,5^. For immunofluorescent staining, the tissue sections were rehydrated and labeled with primary antibodies, including Beclin-1, Lcn2, α-SMA, E-cadherin, Snail, p-Histone H3, Cleaved caspase 3, and then exposed to Texas red-labeled or FITC green-labeled secondary antibodies. Slides were viewed with a Nikon Eclipse 80i microscope equipped with a digital camera (DS-Ri1, Nikon, Shanghai, China).

### Assessment of tubular injury

For general histology, sections were stained with Periodic acid-Schiff (PAS)^5^. The degree of tubular injury was determined using a semiquantitative grade scale that tubular injury was scored on a scale from 0 to 3, where 0 = normal, 1 = injury <30%, 2 = injury 30–60%, 3 = injury >60%. Two sections were randomly selected from each sample of at least three for every group and 10 fields were randomly selected at a magnification of ×200 from each section in PAS staining. At last, an average score was calculated and then made into column diagram.

### Statistical analysis

All the experiments were conducted at least three times. Data depicted in graphs represent the means ± SEM for each group. Intergroup comparison was made using one-way analysis of variance. Multiple means were compared using Tukey’s test. The differences between two groups were determined by Student’s *t*-test. Statistical significant difference between mean values was marked in each graph. *P* < 0.05 was considered significant. The statistical analyses were conducted by using IBM SPSS Statistics 20.0 (Version X; IBM, Armonk, NY, USA).

## Supplementary information

Supplementary Figure 1

Supplementary Figure 2

Supplementary Figure Legends

## References

[1] Johnson RJ (2018). Hyperuricemia, acute and chronic kidney disease, hypertension, and cardiovascular disease: report of a scientific workshop organized by the national kidney foundation. Am. J. Kidney Dis..

[2] Isaka Y, Takabatake Y, Takahashi A, Saitoh T, Yoshimori T (2016). Hyperuricemia-induced inflammasome and kidney diseases. Nephrol. Dial. Transpl..

[3] Suliman ME (2006). J-shaped mortality relationship for uric acid in CKD. Am. J. Kidney Dis..

[4] Liu N (2015). EGF receptor inhibition alleviates hyperuricemic nephropathy. J. Am. Soc. Nephrol..

[5] Bao J (2018). Pharmacological inhibition of autophagy by 3-MA attenuates hyperuricemic nephropathy. Clin. Sci..

[6] Tao M (2019). Blockade of ERK1/2 by U0126 alleviates uric acid-induced EMT and tubular cell injury in rats with hyperuricemic nephropathy. Am. J. Physiol. Ren. Physiol..

[7] Lovisa S (2015). Epithelial-to-mesenchymal transition induces cell cycle arrest and parenchymal damage in renal fibrosis. Nat. Med..

[8] Yang L, Besschetnova TY, Brooks CR, Shah JV, Bonventre JV (2010). Epithelial cell cycle arrest in G2/M mediates kidney fibrosis after injury. Nat. Med..

[9] Crosio C (2002). Mitotic phosphorylation of histone H3: spatio-temporal regulation by mammalian Aurora kinases. Mol. Cell. Biol..

[10] Yang L, Besschetnova TY, Brooks CR, Shah JV, Bonventre JV (2010). Epithelial cell cycle arrest in G2/M mediates kidney fibrosis after injury. Nat. Med..

[11] Pérez-Cadahía B, Drobic B, Davie JR (2009). H3 phosphorylation: dual role in mitosis and interphase. Biochem. Cell Biol..

[12] Hendzel MJ (1997). Mitosis-specific phosphorylation of histone H3 initiates primarily within pericentromeric heterochromatin during G2 and spreads in an ordered fashion coincident with mitotic chromosome condensation. Chromosoma.

[13] Meng X-M, Nikolic-Paterson DJ, Lan HY (2016). TGF-β: the master regulator of fibrosis. Nat. Rev. Nephrol..

[14] Huang S, Susztak K (2016). Epithelial plasticity versus EMT in kidney fibrosis. Trends Mol. Med..

[15] Parrish AR (2017). Matrix metalloproteinases in kidney disease: role in pathogenesis and potential as a therapeutic target. Prog. Mol. Biol. Transl. Sci..

[16] Sampieri CL, Orozco-Ortega RA (2018). Matrix metalloproteinases and tissue inhibitors of metalloproteinases in chronic kidney disease and acute kidney injury: a systematic review of the literature. Hippokratia.

[17] Choe J-Y, Park K-Y, Kim S-K (2015). Oxidative stress by monosodium urate crystals promotes renal cell apoptosis through mitochondrial caspase-dependent pathway in human embryonic kidney 293 cells: mechanism for urate-induced nephropathy. Apoptosis.

[18] Che R, Yuan Y, Huang S, Zhang A (2014). Mitochondrial dysfunction in the pathophysiology of renal diseases. Am. J. Physiol. Ren. Physiol..

[19] Huang MLH (2019). The role of the antioxidant response in mitochondrial dysfunction in degenerative diseases: cross-talk between antioxidant defense, autophagy, and apoptosis. Oxid. Med. Cell Longev..

[20] Sanz AB, Santamaría B, Ruiz-Ortega M, Egido J, Ortiz A (2008). Mechanisms of renal apoptosis in health and disease. J. Am. Soc. Nephrol..

[21] Choi AMK, Ryter SW, Levine B (2013). Autophagy in human health and disease. N. Engl. J. Med..

[22] Huber TB (2012). Emerging role of autophagy in kidney function, diseases and aging. Autophagy.

[23] Livingston MJ (2019). Clearance of damaged mitochondria via mitophagy is important to the protective effect of ischemic preconditioning in kidneys. Autophagy.

[24] Tang C (2018). PINK1-PRKN/PARK2 pathway of mitophagy is activated to protect against renal ischemia-reperfusion injury. Autophagy.

[25] Wang Y, Cai J, Tang C, Dong Z (2020). Mitophagy in acute kidney injury and kidney repair. Cells.

[26] Livingston MJ (2016). Persistent activation of autophagy in kidney tubular cells promotes renal interstitial fibrosis during unilateral ureteral obstruction. Autophagy.

[27] Xu Y (2013). Autophagy and apoptosis in tubular cells following unilateral ureteral obstruction are associated with mitochondrial oxidative stress. Int. J. Mol. Med..

[28] Galluzzi L, Bravo-San Pedro JM, Levine B, Green DR, Kroemer G (2017). Pharmacological modulation of autophagy: therapeutic potential and persisting obstacles. Nat. Rev. Drug Discov..

[29] Wu YT (2010). Dual role of 3-methyladenine in modulation of autophagy via different temporal patterns of inhibition on class I and III phosphoinositide 3-kinase. J. Biol. Chem..

[30] Yang X (2020). WNT1-inducible signaling protein-1 mediates TGF-beta1-induced renal fibrosis in tubular epithelial cells and unilateral ureteral obstruction mouse models via autophagy. J. Cell. Physiol..

[31] Gu J (2019). Calcimimetic compound NPS R-467 protects against chronic cadmium-induced mouse kidney injury by restoring autophagy process. Ecotoxicol. Environ. Saf..

[32] Xue L (2019). Liraglutide promotes autophagy by regulating the AMPK/mTOR pathway in a rat remnant kidney model of chronic renal failure. Int. Urol. Nephrol..

[33] Yang Y, Zhou W, Wang Y, Zhou R (2019). Gender-specific association between uric acid level and chronic kidney disease in the elderly health checkup population in China. Ren. Fail..

[34] Kumagai T (2017). Time to target uric acid to retard CKD progression. Clin. Exp. Nephrol..

[35] Wang H, Li QF, Chow HY, Choi SC, Leung YC (2020). Arginine deprivation inhibits pancreatic cancer cell migration, invasion and EMT via the down regulation of Snail, Slug, Twist, and MMP1/9. J. Physiol. Biochem..

[36] Liu N (2017). Pharmacologic targeting ERK1/2 attenuates the development and progression of hyperuricemic nephropathy in rats. Oncotarget.

[37] Li H (2016). Atg5-mediated autophagy deficiency in proximal tubules promotes cell cycle G2/M arrest and renal fibrosis. Autophagy.

[38] Chen W, Yan Y, Song C, Ding Y, Du T (2017). Microvesicles derived from human Wharton’s Jelly mesenchymal stem cells ameliorate ischemia-reperfusion-induced renal fibrosis by releasing from G2/M cell cycle arrest. Biochem. J..

[39] Duann P, Lin PH (2017). Mitochondria damage and kidney disease. Adv. Exp. Med. Biol..

[40] Galvan DL, Green NH, Danesh FR (2017). The hallmarks of mitochondrial dysfunction in chronic kidney disease. Kidney Int..

[41] Wang J (2020). Bax inhibitor 1 preserves mitochondrial homeostasis in acute kidney injury through promoting mitochondrial retention of PHB2. Theranostics.

[42] Wang J, Zhu P, Li R, Ren J, Zhou H (2019). Fundc1-dependent mitophagy is obligatory to ischemic preconditioning-conferred renoprotection in ischemic AKI via suppression of Drp1-mediated mitochondrial fission. Redox Biol..

[43] Li G-B (2018). Mitochondrial fission and mitophagy depend on cofilin-mediated actin depolymerization activity at the mitochondrial fission site. Oncogene.

[44] Zeng W, Zhang W, Lu F, Gao L, Gao G (2017). Resveratrol attenuates MPP(+)-induced mitochondrial dysfunction and cell apoptosis via AKT/GSK-3beta pathway in SN4741 cells. Neurosci. Lett..

[45] Rizwan H, Pal S, Sabnam S, Pal A (2020). High glucose augments ROS generation regulates mitochondrial dysfunction and apoptosis via stress signalling cascades in keratinocytes. Life Sci..

[46] Haryono A, Nugrahaningsih DAA, Sari DCR, Romi MM, Arfian N (2018). Reduction of serum uric acid associated with attenuation of renal injury, inflammation and macrophages M1/M2 ratio in hyperuricemic mice model. Kobe J. Med. Sci..

[47] Chen YS, Chen CJ, Yan W, Ge HM, Kong LD (2017). Anti-hyperuricemic and anti-inflammatory actions of vaticaffinol isolated from *Dipterocarpus alatus* in hyperuricemic mice. Chin. J. Nat. Med..

[48] Kimura T, Isaka Y, Yoshimori T (2017). Autophagy and kidney inflammation. Autophagy.

[49] Liu N, Shi Y, Zhuang S (2016). Autophagy in chronic kidney diseases. Kidney Dis..

[50] Wang Y (2018). PINK1/Parkin-mediated mitophagy is activated in cisplatin nephrotoxicity to protect against kidney injury. Cell Death Dis..

[51] Jiang M (2012). Autophagy in proximal tubules protects against acute kidney injury. Kidney Int..

[52] Yang D (2018). Autophagy in diabetic kidney disease: regulation, pathological role and therapeutic potential. Cell. Mol. Life Sci..

[53] Johnson RJ (2013). Uric acid and chronic kidney disease: which is chasing which?. Nephrol. Dial. Transpl..

[54] Mizumura K (2014). Mitophagy-dependent necroptosis contributes to the pathogenesis of COPD. J. Clin. Invest..

[55] Basit F (2017). Mitochondrial complex I inhibition triggers a mitophagy-dependent ROS increase leading to necroptosis and ferroptosis in melanoma cells. Cell Death Dis..

[56] Fan S (2019). Hyperuricemia and its related histopathological features on renal biopsy. BMC Nephrol..

[57] Galluzzi L, Bravo-San Pedro JM, Levine B, Green DR, Kroemer G (2017). Pharmacological modulation of autophagy: therapeutic potential and persisting obstacles. Nat. Rev. Drug Discov..

[58] Morita M (2017). mTOR controls mitochondrial dynamics and cell survival via MTFP1. Mol. Cell.

[59] Liu C-W (2018). PM(2.5)-induced oxidative stress increases intercellular adhesion molecule-1 expression in lung epithelial cells through the IL-6/AKT/STAT3/NF-κB-dependent pathway. Part. Fibre Toxicol..

[60] Shih JH (2019). Autophagy inhibition plays a protective role against 3, 4-methylenedioxymethamphetamine (MDMA)-induced loss of serotonin transporters and depressive-like behaviors in rats. Pharmacol. Res..

[61] Zhan L (2018). The roles of autophagy in acute lung injury induced by myocardial ischemia reperfusion in diabetic rats. J. Diabetes Res..

[62] Zhou X (2016). Enhancer of zeste homolog 2 inhibition attenuates renal fibrosis by maintaining Smad7 and phosphatase and tensin homolog expression. J. Am. Soc. Nephrol..

