# Supplementary material for: Delayed treatment with an autophagy inhibitor 3-MA alleviates the progression of hyperuricemic nephropathy
Source: Cell Death Dis. 2020 Jun 17;11(6):467. doi: 10.1038/s41419-020-2673-z (PMC7298642; doi:10.1038/s41419-020-2673-z)
Supplement: Supplementary file 3 — Supplementary Figure Legends [file 41419_2020_2673_MOESM3_ESM.docx]

**Supplemental Figure 1. Inhibition of autophagy regulates the expression of MMP2 and MMP9 in hyperuricemic rats**

The kidney tissue lysates were subjected to immunoblot analysis with specific antibodies against MMP2, MMP9 and GAPDH (**A**). Expression levels of MMP2 (**B**) and MMP9 (**C**) were quantified by densitometry and normalized with GAPDH. Photomicrographs illustrating immunohistochemistry staining of MMP2 (**D**) and MMP9 (**E**). Data are represented as the mean ± SEM (n=6). Means with different superscript letters are significantly different from one another (*P*< 0.05). All scale bars = 20 μm.

Supplemental Figure 2. **The mechanisms by which delayed treatment with 3-MA alleviates the progression of hyperuricemic nephropathy**

Autophagy inhibition is the main mechanism for 3-MA-elicited a series of renoprotective effects. Autophagy inhibition by 3-MA reduces TGF-β/Smad3 pathway-induced EMT and G2/M phase cell cycle arrest of renal epithelial cells, and attenuates STAT3/NF-κB pathway-elicited inflammation effect. In addition, 3-MA also suppresses mitochondrial fission-induced apoptosis of renal tubular epithelial cells. Collectively, autophagy inhibition protects kidney against fibrosis and tubular injury in hyperuricemic nephropathy, leading to improve renal function.
